# PDGF-D promotes cell growth, aggressiveness, angiogenesis and EMT transformation of colorectal cancer by activation of Notch1/Twist1 pathway

**DOI:** 10.18632/oncotarget.14283

**Published:** 2016-12-27

**Authors:** Jinhuang Chen, Wenzheng Yuan, Liang Wu, Qiang Tang, Qinghua Xia, Jintong Ji, Zhengyi Liu, Zhijun Ma, Zili Zhou, Yifeng Cheng, Xiaogang Shu

**Affiliations:** ^1^ Department of Gastrointestinal Surgery, Union Hospital, Tongji Medical College, Huazhong University of Science and Technology, Wuhan, China

**Keywords:** PDGF-D, colorectal cancer, EMT, angiogenesis

## Abstract

Platelet-derived growth factor-D (PDGF-D) plays a crucial role in the progression of several cancers. However, its role in colorectal cancer (CRC) remains unclear. Our study showed that PDGF-D was highly expressed in CRC tissues and was positively associated with the clinicopathological features. Down-regulation of PDGF-D inhibited the tumor growth, migration and angiogenesis of SW480 cells *in vitro* and *in vivo*. Whereas up-regulation of PDGF-D promoted the malignant behaviors of HCT116 cells. Moreover, PDGF-D up-regulated the expression of Notch1 and Twist1 in CRC cells. In addition, PDGF-D expression promoted Epithelial to mesenchymal transition (EMT), which was accompanied with decreased E-cadherin and increased Vimentin expression. Consistently, PDGF-D, Notch1, and Twist1 are obviously up-regulated in transforming growth factor-beta 1 (TGF-β1) treated HCT116 cells. Since Notch1 and Twist1 play an important role in EMT and tumor progression, we examined whether there is a correlation between Notch1 and Twist1 in EMT status. Our results showed that up-regulation of Notch1 was able to rescue the effects of PDGF-D down-regulation on Twist1 expression in SW480 cells, whereas down-regulation of Notch1 reduced Twist1 expression in HCT116 cells. Furthermore, we found that Twist1 promoted EMT and aggressiveness of CRC cells. These results suggest that PDGF-D promotes tumor growth and aggressiveness of CRC, moreover, down-regulation of PDGF-D inactivates Notch1/Twist1 axis, which could reverse EMT and prevent CRC progression.

## INTRODUCTION

Growth factors and their receptors are important in tumor growth, metastasis and angiogenesis in several cancers [1, 2]. PDGF-D belongs to the PDGF family (PDGF-A, B, C, and D) and plays a significant role in cancer progression [3]. PDGF-D is recently discovered and was found to exert its functions through binding to its receptor (PDGFR-β), leading to rapid phosphorylation of PDGFR-β and consequent activation of many downstream signaling pathways [4]. Previous studies revealed that PDGF-D is over-expressed in human breast, pancreatic and gastric cancer and involved in cell growth, aggressiveness, and angiogenesis [3–5]. However, the precise role of PDGF-D in CRC is still unknown.

It has been reported that PDGF-D increases tumor growth and aggressiveness by activating Notch1 and NF-κB in human pancreatic and breast cancer [3, 4]. Liu *et al*. reported that PDGF-D facilitates tumor metastasis by increasing C-X-C chemokine receptor type 4 (CXCR4) expression [6]. Moreover, PDGF-D enhances tumor metastasis and angiogenesis of renal cancer through increasing the expression of matrix metalloproteinase-9 (MMP9) and vascular endothelial growth factor (VEGF) [7]. In addition, PDGF-D plays an important role in EMT transformation [8]. These findings indicate that PDGF-D promotes the development of the human cancers, and it is important to investigate the potential role of PDGF-D in CRC.

It is well established that Notch1 is involved in cell growth, metastasis [9]. Several studies reported that Notch1 is highly expressed in CRC and is tightly linked to CRC progression by regulating the expression of several downstream mediators such as MMP9, VEGF, extracellular signal-related kinase (ERK) [9, 10]. Moreover, PDGF-D promotes tumorigenesis and aggressiveness by activating Notch1 pathway in breast and pancreatic cancers [3, 4], but it is unclear whether this also occurs in CRC. It is well known that EMT is important in tumor invasion and metastasis [8]. Recently, many signaling pathways and growth factors have been found to induce EMT, such as Wnt, TGF-β1, the mammalian Target of Rapamycin (mTOR), and Notch1 [8, 11]. For example, Notch1 over-expression induces EMT by increasing the expression of Vimentin and N-cadherin in pancreatic cancer [12]. Moreover, Notch1 promotes EMT by regulating EMT-transcription factors such as Twist1 in CRC [13]. Thus, further investigation is warranted to identify whether there is a cross-talk between PDGF-D, Notch1, and Twist1 and whether this axis regulates EMT in CRC.

In the present study, we explored the consequence of PDGF-D down/up-regulation in SW480 or HCT116 cells, respectively. Cell growth, aggressiveness characteristics and angiogenesis were evaluated *in vivo* and *in vitro*. The results showed that PDGF-D down-regulation inhibited cell growth, aggressiveness and EMT through the suppression of Notch1/Twist1 axis. Collectively, our study indicates that PDGF-D down-regulation could be an effective approach to treat CRC.

## RESULTS

### PDGF-D is highly expressed in CRC tissues and cell lines

**Figure 1:**
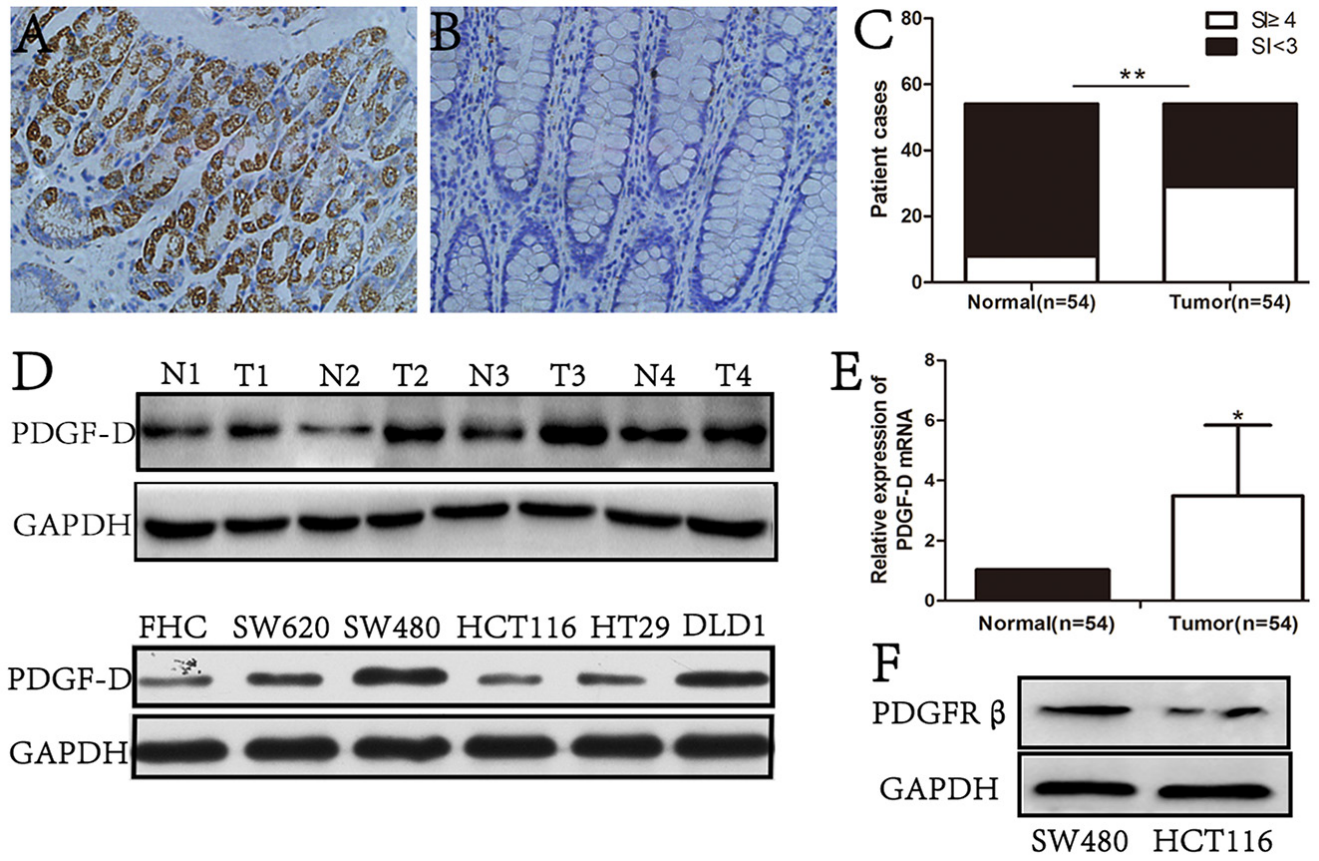
PDGF-D was high expression in CRC tissues and cell lines **A**. immunohistochemical staining showed that the expression of PDGF-D was strong in cytoplasm of tumor cells, but was weak in adjacent noncancerous tissues **B**. Magnification, ×200. **C**. 53.7%(29/54, SI≥4) CRC tissues were positive for PDGF-D expression, while 14.8%(8/54, SI<3) adjacent tissues were positive staining for PDGF-D. **D**. Western blot showed the higher expression of PDGF-D in CRC tissues and cancer cell lines than those in normal tissues. N, normal tissue; T, CRC tissue. **E**. RT-PCR showed that the mRNA of PDGF-D was higher in CRC tissues. **F**. PGGFR-β expression in SW480 and HCT116 cells was detected by western blot. *P<0.05, **P<0.001.

PDGF-D expression was examined in 54 primary CRC tissues, their corresponding normal adjacent mucosa, and cultured FHC, SW620, SW480, HCT116, HT29, DLD1 cell lines. An intense and diffuse cytoplasmic staining pattern of PDGF-D was detected in CRC specimens (Figure 1A) by IHC, whereas the corresponding normal mucosa showed no or weak staining of PDGF-D (Figure 1B and 1C). Furthermore, PDGF-D over-expression was positively correlated with tumor stage (P= 0.02), lymph node stage (P= 0.04), and tumor differentiation (P= 0.03), but not with the age of patients (Table 1). In addition, as shown in Figure 1D, PDGF-D expression was higher in CRC tissues than the corresponding adjacent tissues. PDGF-D was also expressed in all CRC cell lines, but at variable degree. SW480 and DLD1 cells showed higher expression, while HCT116 and FHC cells showed relative lower expression of PDGF-D. Notably, consistent with PDGF-D expression, PDGF-β was highly expressed in SW480 cells compared to HCT116 cells (Figure 1F).

**Table 1:** Clinicopathological characteristics of patients

Clinic pathological factors		n	PDGF-D expression		P
			Positive	Negative	
Age(years)	>60	23	14	9	0.36
	<60	31	15	16	
Gender	Male	35	21	14	0.21
	Female	19	8	11	
Pathologic T stage	T1+T2	21	7	14	0.02
	T3+T4	33	22	11	
Pathologic N stage	N0	18	6	12	0.04
	N1+N2	36	23	13	
Tumor differentiation	Well	12	3	9	0.03
	Moderate	31	19	12	
	Poor	11	7	4	

### PDGF-D expression promotes cell growth and colony formation in CRC cell lines

**Figure 2:**
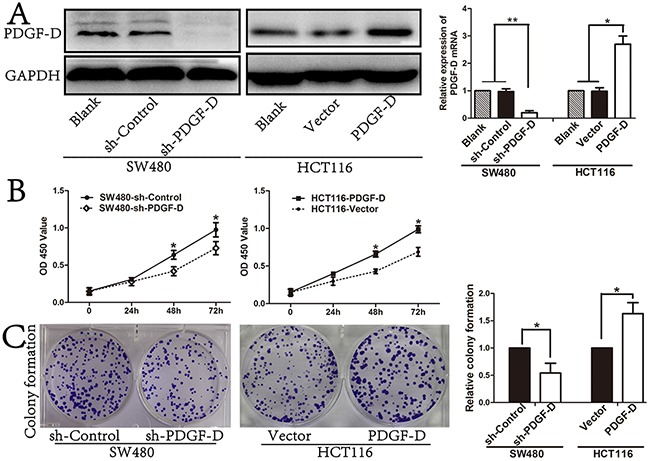
PDGF-D expression promotes cell cell growth and colony formation **A**. Efficacy of PDGF-D silencing or transfection was assessed by western blot and RT-PCR. **B**. Effects of PDGF-D expression on cell growth was tested by CCK8 assay and on capacity of colony formation **C**. *P<0.05, **P<0.001.

PDGF-D/PDGFR-β signaling pathway plays crucial roles in progression of several cancers. To investigate the effect of PDGF-D silencing on cell growth, a lentiviral vector with PDGF-D shRNA was transfected into SW480 cells. Western blot and RT-PCR revealed that shPDGF-D obviously decreased the expression of PDGF-D in SW480 (Figure 2A). Moreover, down-regulation of PDGF-D inhibited the cell growth (Figure 2B) and colony formation (Figure 2C) of SW480 cells compared to the control cells.

In order to further determine the effects of PDGF-D in CRC cells, lentiviral vector with PDGF-D cDNA was transfected into HCT116 cells to investigate the function of PDGF-D. The PDGF-D cDNA transfection significantly increased the expression of PDGF-D in HCT116 (Figure 1A). Subsequently, as shown in Figure 2B and 2C, over-expression of PDGF-D in HCT116 cells obviously increased cell proliferation and colony formation.

### PDGF-D expression promotes cell cycle distribution, aggressiveness, and angiogenesis, but not apoptosis in CRC cell lines

**Figure 3:**
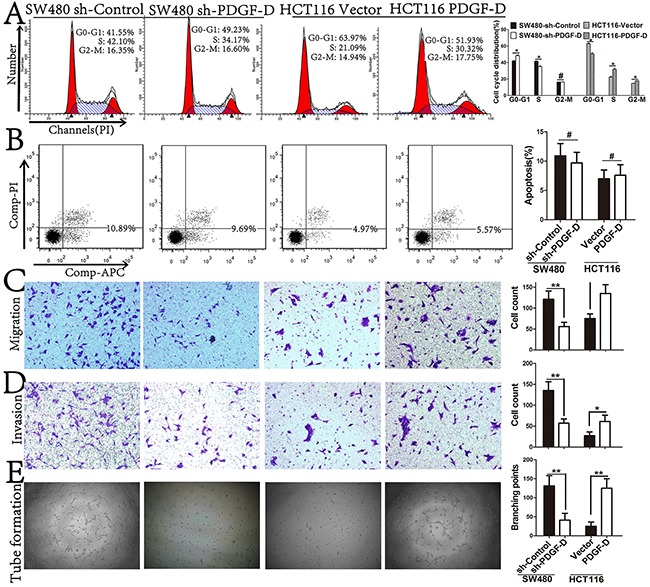
PDGF-D expression promotes cell cycle distribution, aggressiveness, and angiogenesis, but not in apotosis **A and B**. Effects of PDGF-D expression on cell cycle distribution and apoptosis were detected by FACS. **C and D**. Effects of PDGF-D expression on cell migration and invasion were performed by transwell assay. Magnification, ×200. **E**. The tube formations were performed with HUVEC cells treated with conditioned medium. Magnification, ×40. *P<0.05.

As shown in Figure 3A, PDGF-D silencing elevated the percentage of cells at G0/G1 phase, while over-expression of PDGF-D reduced the percentage of cells at G0/G1 phase in CRC cells. However, in apoptosis assay, PDGF-D did not influence the apoptosis rate of CRC cells (Figure 3B). Next, transwell assay was performed to determine whether PDGF-D has any effects on the aggressiveness of CRC cells. Compared to the control cells, PDGF-D silencing decreased the migration and invasion capacity in SW480 cells, while over-expression of PDGF-D increased the aggressiveness in HCT116 cells (Figure 3C and 3D).

To identify the role of PDGF-D on angiogenesis in CRC cells, tube formation assay was performed. Compared to the control cells, the tube formation of HUVECs was decreased upon treatment with conditioned medium from PDGF-D shRNA transfected SW480 cells. Conversely, the tube formation was increased in HCT116 cells (Figure 3E). These results revealed that PDGF-D increased the cell growth, aggressiveness and angiogenesis in CRC cells.

### PDGF-D increases the expression of Notch1 in CRC cells

**Figure 4:**
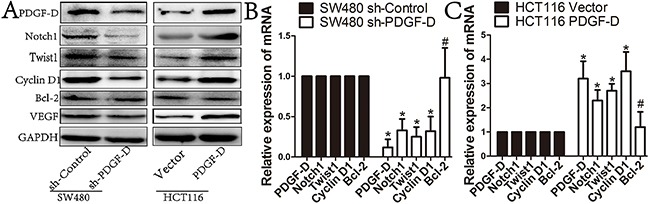
PDGF-D increases the expression of Notch1 in CRC cells **A, B and C**. Western blot and RT-PCR showed that compared to control cells, PDGF-D down-expression resulted in decreased expression of Notch1, Twist1, CyclinD1, and VEGF in SW480 cells, when PDGF-D up-expression increased the expression of the aforementioned proteins in HCT116 cells. But, PDGF-D expression had no effects on expression of Bcl-2. *P<0.05, #P>0.05.

We further explored the mechanism by which PDGF-D regulates cell growth, aggressiveness and angiogenesis in CRC cells. As shown in Figure 4, PDGF-D silencing was found to decrease Notch1 expression in SW480 cells, while PDGF-D up-regulation increased Notch1 expression in HCT116 cells. In addition, the expression of Notch1 downstream genes such as CyclinD1 and VEGF, were obviously decreased upon PDGF-D down-regulation in SW480 cells (Figure 4A and 4B). Conversely, CyclinD1 and VEGF were up-regulated in HCT116 cells (Figure 4A and 4C). However, B-cell lymphoma-2 (bcl-2) expression showed no obvious change in transfected cells compared to the control cells. All these findings were consistent with the results shown in Figure 3 and demonstrated that PDGF-D increases Notch1 expression in CRC cells.

### PDGF-D induces the EMT profile in CRC cells

**Figure 5:**
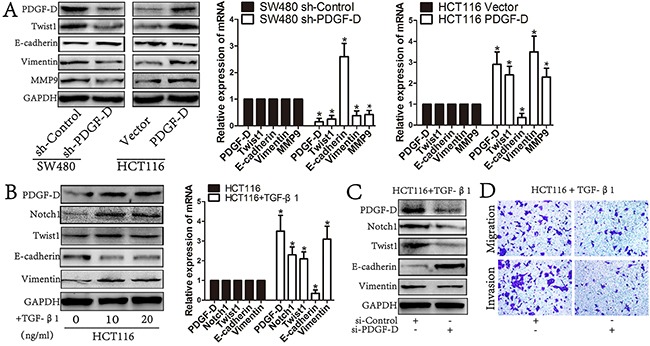
PDGF-D induces the EMT profile in CRC cells **A**. Western blot and RT-PCR revealed that PDGF-D down-expression reversed EMT transformation with decreased expression of Twist1, Vimentin, MMP9 and increased E-cadherin. Conversely, PDGF-D up-expression promoted EMT transformation in CHT116 cells. **B**. The expression of PDGF-D was significantly increased in TGF-β1 treated HCT116 cells involving EMT transformation with increased expression of Twist1, Vimentin and decreased E-cadherin. **C**. Western blot showed that inhibition of PDGF-D increased the expression of E-cadherin and decreased the expression of Vimentin, MMP9, Twist1 and Notch1 in TGF-β1 treated HCT116 cells. **D**. Inhibition of PDGF-D led to decreased migration and invasion in TGF-β1 treated HCT116 cells. Magnification, ×200. *P<0.05.

To investigate whether PDGF-D could promote EMT in CRC cells, PDGF-D stable knockdown SW480 cells and HCT116 cells that are stably expressing PDGF-D were established. Western blot and RT-PCR showed that the PDGF-D silencing down-regulated the expression of Twist1 in SW480 cells, as well as Vimentin and MMP9, whereas E-cadherin was increased (Figure 5A). On the contrary, in HCTT16 cells, PDGF-D promoted EMT with increased Twist1, Vimentin, and MMP9 while E-cadherin was decreased (Figure 5A). Together, these data suggests that PDGF-D promotes EMT transformation in CRC cells.

### PDGF-D is significantly increased in TGF-β1 treated HCT116 cells

HCT116 cells treated with human TGF-β1 (10ng/mL, 20ng/mL) for 72h has shown elevated EMT profile as well as PDGF-D increased expression (Figure 5B). Notably, TGF-β1 treated HCT116 cells showed obvious EMT profiles with decreased E-cadherin, increased Vimentin and Twist1 (Figure 5B). Meanwhile, the expression of PDGF-D and Notch1 were increased in TGF-β1 treated HCT116 cells. These results demonstrate that PDGF-D elevated expression was correlated with active EMT in CRC cells.

### Downregulation of PDGF-D reversed EMT in TGF-β1 treated HCT116 cells

To further confirm whether up-regulated PDGF-D contributed to EMT of CRC cells, siPDGF-D was transfected into TGF-β1 treated HCT116 cells to examine whether down regulation of PDGF-D could reverse the EMT in those cells. After incubated with siPDGF-D for 48h, the TGF-β1 treated cells showed a Mesenchymal to Epithelial transition (MET)-like transformation with increased E-cadherin, as well as decreased Vimentin, Twist1 and Notch1 (Figure 5C). Moreover, compared to the parent cells, the capacity of migration and invasion was decreased in TGF-β1 treated HCT116 cells with siPDGF-D transfection (Figure 5D).

### PDGF-D promotes cell growth, aggressiveness and EMT transformation of CRC through activation of Notch1/Twist1 pathway

**Figure 6:**
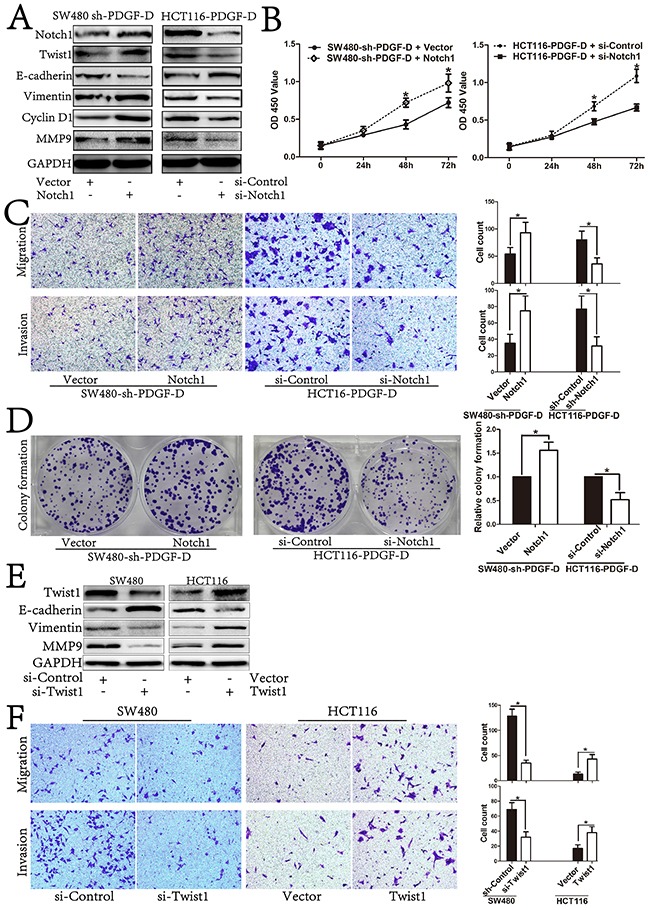
PDGF-D promotes cell growth, aggressiveness and EMT transformation of CRC by activation of Notch1/Twist1 pathway **A**. Western blot showed restoration of Notch1 led to increased expression of Twist1, CyclinD1, Vimentin, MMP9 and decreased expression of E-cadherin in sh-PDGF-D transfected SW480 cells. Conversely, inhibition of Notch1 led to decreased expression of Twist1, CyclinD1, Vimentin, MMP9 and increased expression of E-cadherin in PDGF-D cDNA transfected HCT116 cells. **B**. Effects of Notch1 up/down-expression on cell growth, migration and invasion **C**., and capacity of colony formation in sh-PDGF-D transfected SW480 cells and PDGF-D cDNA transfected HCT116 cells, respectively **D**, **E**. Effects of Twist1 on EMT transformation and aggressiveness **F**. of CRC cells. Magnification, ×200. *P<0.05.

Since our previous results showed that PDGF-D expression was positively correlated with the expression of Notch1 and Twist1 (Figure 4), we explored whether Notch1 could be involved in PDGF-D-mediated regulation of Twist1. As shown in Figure 6A, Notch1 over-expression by cDNA transfection rescued shPDGF-D-mediated down-regulation of Twist1 and MET transformation with decreased E-cadherin, increased Vimentin and MMP9 in SW480 cells. Moreover, Notch1 over-expression rescued the inhibition of cell migration and invasion in SW480-shPDGF-D cells (Figure 6C). Conversely, Notch1 down-regulation by siNotch1 inhibited the PDGF-D cDNA-mediated up-regulation of Twist1 and EMT transformation in HCT116 cells with concurrent increase in E-cadherin expression, and decreased Vimentin and MMP9 expression (Figure 6A). Similarly, Notch1 down-regulation negated the aggressiveness effects of PDGF-D up-regulation in HCT116 cells (Figure 6C).

In addition, Notch1 over-expression rescued the inhibitory effect of cell proliferation and colony formation in SW480-shPDGF-D cells (Figure 6B and 6D), while Notch1 down-regulation counteracted the proliferative effects of PDGF-D up-regulation in HCT116 cells (Figure 6B and 6D).

To further clarify that PDGF-D influenced EMT profile of CRC cells via regulating the expression of Twist1, si-Twist1 and Twist1 cDNA were transfected into CRC cells to test the effects of Twist1 on EMT. As shown in Figure 6E, Twist1 silencing inhibited EMT transformation with increased E-cadherin, while Vimentin and MMP9 were decreased in SW480 cells. On the contrary, Twist1 up-regulation promoted EMT transformation. Furthermore, Twist1 silencing inhibited the migration and invasion capacity of SW480 cells, whereas Twist1 up-regulation increased the aggressiveness of HCT116 cells (Figure 6F). Together, these findings further indicate the mechanistic involvement of Notch1/Twist1 axis in PDGF-D-mediated role in cell growth, aggressiveness and EMT of CRC cells.

### PDGF-D promotes tumorigenesis, angiogenesis and EMT profile of CRC cells *in vivo*

**Figure 7:**
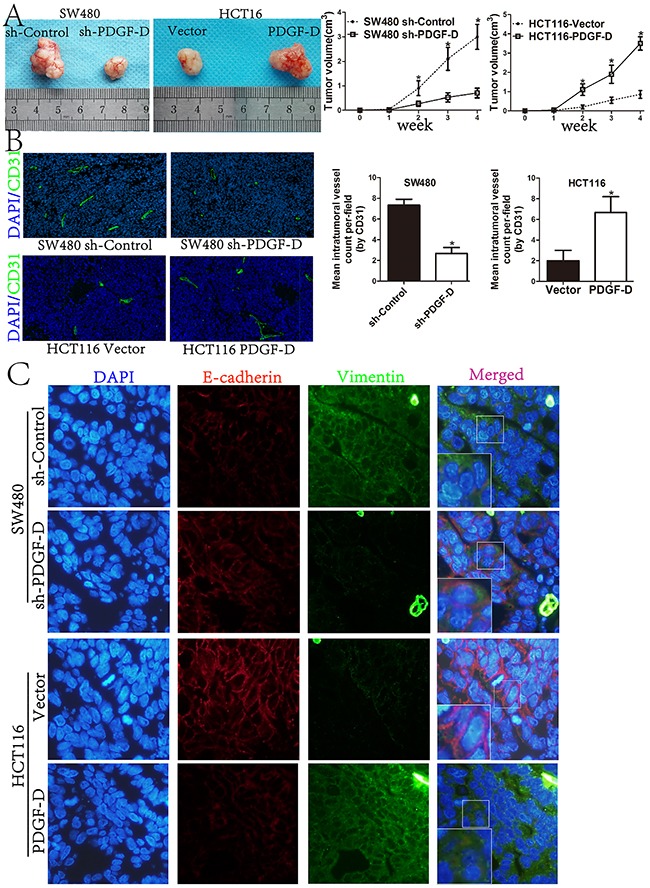
PDGF-D promotes tumorigenesis, angiogenesis and EMT profile of CRC cells in vivo **A**. Compared to control cells, the tumor size of sh-PDGF-D transfected SW480 cells was significantly decreased, while the size of PDGF-D cDNA transfected HCT116 cells was increased. **B**. Immunohistofluorescence staining showed that CD31-positive (green) mean vessel density within tumors was decreased after stable transfection of sh-PDGF-D. Conversely, CD31-positive (green) mean vessel density within tumors was increased after stable transfection of PDGF-D cDNA.Magnification, ×40. **C**. Active EMT process in xenograft tumors with double-labeled immunofluorescence for E-cadherin (red) and Vimentin (green), nucleus was stained with DAPI (blue). Magnification, ×400. *P<0.05.

In order to investigate the roles of the endogenous PDGF-D on tumorigenesis *in vivo*, stable transfected SW480 or HCT116 cells, were injected into the flank of male Balb/c nu/nu mice. As shown in Figure 7A, compared to the control cells, PDGF-D silencing in SW480 cells reduced the volume of subcutaneous xenograft tumors. Conversely, over-expression of PDGF-D in HCT116 increased the tumor volume. Moreover, the immunohistofluorescence staining showed that CD31-positive vessel density was decreased within tumors with shPDGF-D transfection, while PDGF-D increased the vessel density (Figure 7B). The findings are consistent with our results *in vitro*, indicating that over-expression of PDGF-D promoted cell growth and angiogenesis.

To further confirm the EMT profile in subcutaneous xenograft tumors, double labeled immunohistofluorescence staining for E-cadherin and Vimentin was performed. Compared to control cells, E-cadherin staining was increased, while Vimentin staining was decreased in xenograft tumors with shPDGF-D transfection. Conversely, E-cadherin was down-regulated in PDGF-D cDNA transfected HCT116 cells, while Vimentin was increased (Figure 7C).

## DISCUSSION

CRC remains the third most common cause of cancer-related morbidity and mortality worldwide. Although the comprehensive therapy for CRC has been improved significantly; the prognosis of CRC patients remains poor [14]. Thus, exploring novel biomarkers is essential for further biological targeted therapy in CRC.

Previous studies revealed that PDGF-D exerts its functions by activating several downstream signalings including Notch1/NF-κB, mitogen-activated protein kinase (MAPK), CXCR4, phosphatidylinositol 3-kinase (PI3K), ERK, and mTOR signaling pathways to facilitate its oncogenic functions [4, 15]. However, the roles and precise mechanisms of PDGF-D on tumor growth, aggressiveness, and angiogenesis in CRC remains unclear. Our study showed that PDGF-D was highly expressed in CRC tissues and it could promote tumor growth, invasion, angiogenesis, and EMT transformation by activating Notch1/Twist1 axis.

PDGF-D is important in the progression of several human cancers, such as breast, gastric, and prostate cancers [16, 17]. However, the expression profile and specific functions of PDGF-D in CRC have not been previously investigated. Our study revealed PDGF-D was highly expressed in CRC tissues and was positively correlated with TNM stage. Overall survival (OS) analysis was not performed due to the lack of related data. Furthermore, PDGFR-β is highly expressed in CRC tissues and it is also associated with tumor growth and metastasis in CRC [18, 19].

The cellular functions and mechanisms of PDGF-D were investigated and showed that PDGF-D promoted tumor growth, colony formation and angiogenesis in CRC cells. Inhibiting PDGF-D expression was found to arrest cells at G0/G1 phase, while over-expression of PDGF-D reduced the percentage of cells at G0/G1 phase. Moreover, PDGF-D positively regulated the expression of CyclinD1 and VEGF in CRC cells. These results suggested that PDGF-D could regulate tumor growth of CRC by affecting cell cycle distribution and vessel formation. Our findings are consistent with the previous studies in several human cancers [3, 5].

Distant metastasis is one of the leading causes of death in patients with CRC [20]. Our study revealed that over-expression of PDGF-D promoted the capacity of cell migration and invasion of CRC cells. Previous studies showed PDGF-D facilitated tumor invasion and metastasis by activating Notch1, PI3K, ERK, or CXCR4 pathways in breast, lung and brain cancers [4, 6, 21]. These results suggest that PDGF-D expression contributes to tumor growth, aggressiveness, and angiogenesis in CRC.

Notch1 signaling plays a key role in tumor progression of several human cancers, including CRC [9]. In the present study, we showed that PDGF-D positively regulated the expression of Notch1 in CRC cells. Moreover, restoration of Notch1 rescued the inhibition of cell proliferation, migration, and invasion in SW480-shPDGF-D cells. Conversely, down-regulation of Notch1 negated the cell growth and aggressiveness effects of HCT116-PDGF-D. Therefore, these results suggest that PDGF-D expression contributes to the tumor growth, aggressiveness and angiogenesis which could be partly mediated via activating Notch1 signaling pathway.

It is believed that EMT is involved in tumor metastasis [8]. Hallmarks of EMT include Slug, Snail and Twist1, which play vital roles in EMT transformation and enhance tumor metastasis. Twist1 is a transcription factor that is involved in progression of EMT and tumor metastasis in CRC [22]. Our study showed that the expression of PDGF-D was positively correlated with Twist1 expression in CRC cells. Meanwhile, we showed that PDGF-D down-regulation reversed EMT, and, conversely, that PDGF-D over-expression induced EMT in CRC cells. To further confirm the role of PDGF-D expression in EMT transformation, HCT116 cells were treated with TGF-β1 to induce EMT transformation. Our results showed that PDGF-D, Notch1 and Twist1 were significantly up-regulated in the TGF-β1 treated cells. Furthermore, PDGF-D silencing was found to reduce the expression of Notch1 and Twist1, and it also reversed the EMT profile in TGF-β1 treated HCT116 cells. These findings indicate that there is a cross-talk between PDGF-D, Notch1, and Twist1 during the acquisition of EMT transformation in CRC cells.

Then, we asked whether PDGF-D promoted EMT via Notch1 mediated Twist1 up-regulation and our results showed that Notch1 over-expression rescued the inhibition of cell migration and invasion in SW480-shPDGF-D cells, while Notch1 down-regulation counteracted the aggressiveness effects of PDGF-D up-regulation in HCT116 cells. Furthermore and as expected, we found that Twist1 expression was reduced as a consequence of PDGF-D down-regulation, which was rescued by Notch1 up-regulation in SW480 cells. In contrast, down-regulation of Notch1 decreased the expression of Twist1 in HCT116 cells. Last, we further conformed that Twist promoted cell aggressiveness and EMT transformation with decreased E-cadherin and increased Vimentin and MMP9 in CRC cells.

In summary, our study indicates that PDGF-D plays a critical role in regulating tumor growth and metastasis in CRC. This effect is mediated by PDGF-D mediated activation of Notch1/Twist1 axis and subsequent reversal of EMT transformation. Thus, targeting PDGF-D is a potential therapeutic approach that could effectively inhibit tumor growth, angiogenesis and metastasis in CRC patients.

## MATERIALS AND METHODS

### Patient tissue sample and ethics statement

The study was approved by the ethics committee of Tongji Medical College, Huazhong University of science and technology, China. A total of 54 pairs of CRC tissue samples including cancer tissues and corresponding normal adjacent mucosa, were collected from Gastrointestinal surgery of Union Hospital (Wuhan, China) from June 2014 to March 2016. All the samples are from patients with colon or rectal cancer who had not received any chemoradiotherapy before operation. The fresh tissues were either snap-frozen in -80°C for further protein or RNA analysis. All samples were obtained with written informed consent of patients and their parents. The clinical data including age, gender, diagnosis, TNM stage, were listed in Table 1.

### Cell culture and reagents

FHC cell line (human colon normal epithelium cell) was cultured in DMEM/F12 (BOSTER, China). CRC cell lines SW620 and SW480 were maintained in L15 (BOSTER, China), while HCT116 and HT29 cell lines were cultured in McCoy’s 5A (BOSTER, China). CRC cell line DLD1 and umbilical vein endothelial cells HUVEC were maintained in RPMI1640 (Hyclone, USA). All cell lines were maintained in complete medium with 10% fetal bovine serum (FBS, Sciencecell, USA), streptomycin (100μg/mL, Sigma, USA) and penicillin (100U/mL, Sigma, USA) in a humidified incubator at 37°C and 5% CO2. Cell apoptosis and cell cycle kits were purchased from AntGene (China). Antibody against PDGF-D, Twist1, Bcl-2, MMP9, CyclinD1, and Vimentin were purchased from abcam (USA), while E-cadherin, and Notch1 were from Santa Cruz (USA). PDGFRβ antibody was from Elabscience (USA).

### Western blot

Tissues or cell protein was extracted using cold RIPA buffer with PMSF (sigma, USA). Equal amount of total proteins were loaded into SDS-PAGE gels and separated by electrophoresis and transferred onto PVDF membranes, which were then incubated with various antibodies. Image J was used to analyze the band density.

### Real-time PCR

The total RNA was isolated from cells or tissues using Trizol (Takara, Japan) and was reversely transcribed to cDNA with RT Master Mix (Takara, Japan) according to the manufactures’ instruction. The RT-PCR was performed with SYBR Master Mix (Takara, Japan) using StepOne-Plus system (ABI, USA). The RT-PCR was performed with 40 cycles under the following conditions: denature at 95°C for 30s, anneal at 60°C for 1min and extend at 95°C for 5s. The relative mRNA level of a gene was normalized to GAPDH and was calculated with 2^-ΔΔCt^ method. The primers of RT-PCR were listed in Table 2.

**Table 2:** Primer sets used for RT-PCR

Primer set	Primers	Sequence(5′-3′)
GAPDH	Forward	GGGGAGCCAAAAGGGTCATCATCT
	Reverse	GACGCCTGCTTCACCACCTTCTTG
PDGF-D	Forward	GTGGAGGAAATTGTGGCTGT
	Reverse	CGTTCATGGTGATCCAACTG
PDGFR-β	Forward	AAGGGACAAAGAGGGCAAAT
	Reverse	AGGTCTGGGCAGTGACAAAA
Notch1	Forward	CGCACAAGGTGTCTTCCAG
	Reverse	CGGCGTGTGAGTTGATGA
Twist1	Forward	CATTCTCAAGAGGTCGTGCCA
	Reverse	CAGGCCAGTTTGATCCCAGTA
E-cadherin	Forward	GCCCTGCCAATCCCGATGAAA
	Reverse	GGGGTCAGTATCAGCCGCT
Vimentin	Forward	GCTTCAGAGAGAGGAAGCCGAAAA
	Reverse	CCGTGAGGTCAGGCTTGGAAA
MMP9	Forward	CAGAGATGCGTGGAGAGT
	Reverse	TCTTCCGAGTAGTTTTGG
VEGF	Forward	CTGACGGACAGACAGACAGACAC
	Reverse	GCCCAGAAGTTGGACGAAAA
Bcl-2	Forward	TGTGTGGAGAGCGTCAAC
	Reverse	ACAGCCAGGAGAAATCAA
CyclinD1	Forward	GAACACGGCTCACGCTTAC
	Reverse	CCCAGACCCTCAGACTTGC

### Lentiviral vector, plasmids, siRNA, and transfection conditions

Lentiviral vector with PDGF-D shRNA or PDGF-D cDNA were from Shanghai GeneChem Co., Ltd (China). Notch1 siRNA, siRNA control and Notch1 cDNA plasmids were purchased from Guangzhou RiboBio Co., Ltd (China). To establish stable cell line with down or up regulation of PDGF-D, lentiviral vector with PDGF-D shRNA or PDGF-D cDNA were transfected into SW480 (SW480-shPDGF-D) and HCT116 (HCT116-PDGF-D) cells, respectively. Colon cancer cells were transfected with Notch1 siRNA or Notch1 cDNA using Lipofectamine 2000. SW480 and HCT116 cells were transfected according to the manufactures’ instructions.

### Cell growth assay (CCK8)

Transfected or control cells (SW480: 4×10^3^/100μL, HCT116: 6×10^3^/100μL) were seeded in a 96-well plate. After 24h, 48h and 72h of incubation, CCK8 (Dojindo, Japan) assay was performed as described previously. The absorbance was detected at 450 nm.

### Colony formation assay

Cell suspension with 500 cells was seeded into a 6-well plate, which was incubated for 2 weeks. The clones were stained with 0.05% crystal violet and were counted under the microscope.

### Cell migration and invasion assays

SW480 (5×10^4^ cells) or HCT116 (4×10^5^ cells) cell suspensions in FBS free medium were seeded into a transwell chamber (BD, USA), which was coated with (invasion) or without (migration) ECM. After 20h incubation, the cells were stained with 0.05% crystal violet for visualization and counted in six random fields.

### Cell apoptosis assay

Cells were collected, washed in phosphate buffered saline (PBS), and incubated with appropriate Annexin-V APC and PI (AntGent, China) for 30min at 37°C. Then, the cells were detected with BD FACS Flow Cytometer (BD, USA).

### Cell cycle assay

Cells in log-phase of growth were harvested, washed in cold PBS, and fixed in 75% cold ethanol. The cells were then incubated with propidium iodide (PI, 50μg/mL, AntGent, China) for 10 min. BD FACS Flow Cytometer (BD, USA) was used to detect the cell cycle distribution.

### Immunohistochemistry (IHC)

Immunohistochemical staining was carried out as described earlier [4]. The IHC result was assessed by two pathologists. To assess the expression of PDGF-D, the procedure was performed as following: 1, assessing the intensity score of the stained tissues (0, negative; +1, weak positive; +2, moderate positive; +3, strong positive); 2, calculating the percentage of positive tumor cells (negative: 0, <10%; weak positive: +1, 11%-25%; moderate positive: +2, 26%-50%; strong positive: +3, >50%); 3, calculating the staining index (SI), SI= (intensity score in 1) × (positive score in 2). SI <3 was classified as low expression, while SI ≥4 was classified as high expression.

### Immunohistofluorescence staining

The immunohistofluorescence staining in paraffin-embedded sections was performed as described previously [5]. The epifluorescence (Olympus, Japan) was used to take the fluorescent images.

### Tube formation assay

A 96-well plate was coated with 50μL growth factor-reduced matrigel. HUVECs were starved in RPMI1640 medium without FBS overnight. The cells were suspended in RPMI1640 medium, which was added with appropriate conditioned medium collected from PDGF-D shRNA, PDGF-D cDNA and empty vector transfected CRC cells. Then, the cell suspension with 2×10^4^ cells was seeded into the 96-well plate and incubated for 8h. Image J was used to measure the branching points and the lengths of the tube walls to assess the activity of angiogenesis.

### Animal experiments

All animal experiments were approved by the Institutional Animal Care and Treatment Committee of Tongji Medical College of Huazhong University of science and technology, China. Transfected cells were collected and suspended in PBS. 5-weeks old male Balb/c nu/nu mice (n= 5 mice per group) were injected subcutaneously with 5 × 10^6^ stably transfected cells. The subcutaneous tumor xenografts size was measured using a caliper every week and was calculated using the following formula: 0.5 × width^2^ × length. Four weeks later, the mice were sacrificed and the subcutaneous tumor xenografts were collected for Immunohistofluorescence staining and angiogenesis.

### Statistical analysis

All results presented in this study were performed at least three times. The data were presented as the mean ± standard deviation (SD). Difference between groups was tested using t test. The χ^2^ analysis was used to compare the expression of PDGF-D and individual clinicopathological features. Value of P<0.05 was considered to be statistically significant.

## References

[1] Kaulfuss S, Seemann H, Kampe R, Meyer J, Dressel R, Konig B, Scharf JG, Burfeind P (2013). Blockade of the PDGFR family together with SRC leads to diminished proliferation of colorectal cancer cells. Oncotarget.

[2] Cunningham D, Humblet Y, Siena S, Khayat D, Bleiberg H, Santoro A, Bets D, Mueser M, Harstrick A, Verslype C, Chau I, Van Cutsem E. (2004). Cetuximab monotherapy and cetuximab plus irinotecan in irinotecan-refractory metastatic colorectal cancer. N Engl J Med.

[3] Ahmad A, Wang Z, Kong D, Ali R, Ali S, Banerjee S, Sarkar FH (2011). Platelet-derived growth factor-D contributes to aggressiveness of breast cancer cells by up-regulating Notch and NF-kappaB signaling pathways. Breast Cancer Res Treat.

[4] Wang Z, Kong D, Banerjee S, Li Y, Adsay NV, Abbruzzese J, Sarkar FH (2007). Down-regulation of platelet-derived growth factor-D inhibits cell growth and angiogenesis through inactivation of Notch-1 and nuclear factor-kappaB signaling. Cancer Res.

[5] Zhao L, Zhang C, Liao G, Long J (2010). RNAi-mediated inhibition of PDGF-D leads to decreased cell growth, invasion and angiogenesis in the SGC-7901 gastric cancer xenograft model. Cancer Biol Ther.

[6] Liu J, Liao S, Huang Y, Samuel R, Shi T, Naxerova K, Huang P, Kamoun W, Jain RK, Fukumura D, Xu L (2011). PDGF-D improves drug delivery and efficacy via vascular normalization, but promotes lymphatic metastasis by activating CXCR4 in breast cancer. Clin Cancer Res.

[7] Xu L, Tong R, Cochran DM, Jain RK (2005). Blocking platelet-derived growth factor-D/platelet-derived growth factor receptor beta signaling inhibits human renal cell carcinoma progression in an orthotopic mouse model. Cancer Res.

[8] Wu Q, Hou X, Xia J, Qian X, Miele L, Sarkar FH, Wang Z (2013). Emerging roles of PDGF-D in EMT progression during tumorigenesis. Cancer Treat Rev.

[9] Espinoza I, Miele L (2013). Deadly crosstalk: Notch signaling at the intersection of EMT and cancer stem cells. Cancer Lett.

[10] Fender AW, Nutter JM, Fitzgerald TL, Bertrand FE, Sigounas G (2015). Notch-1 promotes stemness and epithelial to mesenchymal transition in colorectal cancer. J Cell Biochem.

[11] Wu Q, Wang R, Yang Q, Hou X, Chen S, Hou Y, Chen C, Yang Y, Miele L, Sarkar FH, Chen Y, Wang Z (2013). Chemoresistance to gemcitabine in hepatoma cells induces epithelial-mesenchymal transition and involves activation of PDGF-D pathway. Oncotarget.

[12] Bao B, Wang Z, Ali S, Kong D, Li Y, Ahmad A, Banerjee S, Azmi AS, Miele L, Sarkar FH (2011). Notch-1 induces epithelial-mesenchymal transition consistent with cancer stem cell phenotype in pancreatic cancer cells. Cancer Lett.

[13] Peinado H, Olmeda D, Snail Cano A (2007). Zeb and bHLH factors in tumour progression: an alliance against the epithelial phenotype?. Nat Rev Cancer.

[14] Chen J, Xia Q, Jiang B, Chang W, Yuan W, Ma Z, Liu Z, Shu X (2015). Prognostic Value of Cancer Stem Cell Marker ALDH1 Expression in Colorectal Cancer: A Systematic Review and Meta-Analysis. PLoS One.

[15] Park YK, Jang BC, Choi M (2013). Platelet-derived growth factor-D induces expression of cyclooxygenase-2 in rat mesangial cells through activation of PI3K/PKB and PKCs. Int J Mol Med.

[16] Najy AJ, Won JJ, Movilla LS, Kim HR (2012). Differential tumorigenic potential and matriptase activation between PDGF B versus PDGF D in prostate cancer. Mol Cancer Res.

[17] Ding J, Li XM, Liu SL, Zhang Y, Li T (2014). Overexpression of platelet-derived growth factor-D as a poor prognosticator in endometrial cancer. Asian Pac J Cancer Prev.

[18] Kitadai Y, Sasaki T, Kuwai T, Nakamura T, Bucana CD, Hamilton SR, Fidler IJ (2006). Expression of activated platelet-derived growth factor receptor in stromal cells of human colon carcinomas is associated with metastatic potential. Int J Cancer.

[19] Erben P, Horisberger K, Muessle B, Muller MC, Treschl A, Ernst T, Kahler G, Strobel P, Wenz F, Kienle P, Post S, Hochhaus A, Willeke F (2008). mRNA expression of platelet-derived growth factor receptor-beta and C-KIT: correlation with pathologic response to cetuximab-based chemoradiotherapy in patients with rectal cancer. Int J Radiat Oncol Biol Phys.

[20] Massarweh NN, Chiang YJ, Xing Y, Chang GJ, Haynes AB, You YN, Feig BW, Cormier JN (2014). Association between travel distance and metastatic disease at diagnosis among patients with colon cancer. J Clin Oncol.

[21] LaRochelle WJ, Jeffers M, Corvalan JR, Jia XC, Feng X, Vanegas S, Vickroy JD, Yang XD, Chen F, Gazit G, Mayotte J, Macaluso J, Rittman B (2002). Platelet-derived growth factor D: tumorigenicity in mice and dysregulated expression in human cancer. Cancer Res.

[22] Okada T, Suehiro Y, Ueno K, Mitomori S, Kaneko S, Nishioka M, Okayama N, Sakai K, Higaki S, Hazama S, Hirata H, Sakaida I, Oka M (2010). TWIST1 hypermethylation is observed frequently in colorectal tumors and its overexpression is associated with unfavorable outcomes in patients with colorectal cancer. Genes Chromosomes Cancer.

